# Impact of maternal ART on mother-to-child transmission (MTCT) of HIV at six weeks postpartum in Rwanda

**DOI:** 10.1186/s12889-018-6154-6

**Published:** 2018-11-12

**Authors:** Placidie Mugwaneza, Alexandre Lyambabaje, Aline Umubyeyi, James Humuza, Landry Tsague, Fabian Mwanyumba, Vincent Mutabazi, Sabin Nsanzimana, Muhayimpundu Ribakare, Ange Irakoze, Emmanuel Mutaganzwa, Carl Lombard, Debra Jackson

**Affiliations:** 1 Rwanda Biomedical Center/Institute of HIV, Disease Prevention and Control (RBC/IHDPC), KN 3 Road, Kigali, Rwanda; 2 School of Public Health, College of Medicine and Health Sciences, National University of Rwanda (SPH-CMHS-UR), Kigali, 5229 Rwanda; 3 UNICEF, Western and Central Africa Regional Office, Dakar, Senegal; 4 UNICEF Rwanda, Boulevard de l’Umunganda, Kacyiru, Kigali, Rwanda; 5 National Reference Laboratory Rwanda, KN 3 Road, Kigali, Rwanda; 6 Medical Research Council, South Africa (MRC-SA), Francie van Zyl Drive, Parow, 7505 South Africa; 7 University of the Western Cape, South Africa (UWC-SA), PB X17 Robert Sobukwe Road, Bellville, 7535 South Africa; 8 UNICEF, New York, 3 UN Plaza, New York, NY 10017 USA

**Keywords:** EMTCT, 6 weeks vertical transmission rate, Operational effectiveness, Sentinel survey, Rwanda, MTCT, EMTCT surveillance, Maternal ART

## Abstract

**Background:**

In 2010, Rwanda adopted ART for prevention of mother to child transmission of HIV from pregnant women living with HIV during pregnancy and breasfeeding period. This study examines rates of mother-to-child-transmission of HIV at 6–10 weeks postpartum and risk factors for mother-to-child transmission of HIV (MTCT) among HIV infected women on ART during pregnancy and breastfeeding.

**Methods:**

A cross-sectional survey study was conducted between July 2011–June 2012 among HIV-exposed infants aged 6–10 weeks and their mothers/caregivers. Stratified multi-stage, probability proportional to size and systematic sampling to select a national representative sample of clients. Consenting mothers/caregivers were interviewed on demographic and program interventions. Dry blood spots from HIV-exposed infants were collected for HIV testing using DNA PCR technique. Results are weighted for sample realization. Univariable analysis of socio-demographic and programmatic determinants of early mother-to-child transmission of HIV was conducted. Variables were retained for final multivariable models if they were either at least of marginal significance (*p*-value < 0.10) or played a confounding role (the variable had a noticeable impact > 10% change on the effect estimate).

**Results:**

The study sample was 1639 infants with HIV test results. Twenty-six infants were diagnosed HIV-positive translating to a weighted MTCT estimate of 1.58% (95% CI 1.05–2.37%). Coverage of most elimination of MTCT (EMTCT) program interventions, was above 80, and 90.4% of mother-infant pairs received antiretroviral treatment or prophylaxis. Maternal ART and infant antiretroviral prophylaxis (OR 0.01; 95%CI 0.001–0.17) and maternal age older than 25 years were significantly protective (OR 0.33; 95%CI 0.14–0.78). No disclosure of HIV status, not testing for syphilis during pregnancy and preterm birth were significant risk factors for MTCT. Factors suggesting higher socio-demographic status (flush toilet, mother self-employed) were borderline risk factors for MTCT.

**Conclusion:**

ART for all women during pregnancy and breastfeeding was associated with the estimated low MTCT rate of 1.58%. Mothers who did not receive a full package of anti-retroviral therapy according to the Rwanda EMTCT protocol, and young and single mothers were at higher risk of MTCT and should be targeted for support in preventing HIV infection.

## Background

The global community has committed to the elimination of mother-to-child transmission of HIV (EMTCT) by 2015 and keeping their mothers alive, with targets of < 5% transmission (< 2% for early postnatal) and antiretroviral therapy (ART) coverage of at least 90% [1–4] Measuring impact of EMTCT programs is necessary to track acheivements towards this goal.

Rwanda is located in Eastern Africa, with 10.5 million inhabitants. HIV prevalence in 2017 was estimated at 3.1% in the general population and 3.8% among women age 15–49 years. [5] The national EMTCT program started in 2001, and a national EMTCT scale-up plan (2007–2012) articulated a strategy to deliver comprehensive EMTCT interventions within maternal and child health services. [6] The EMTCT program reached 87% of health facilities by 2012. All services were generally provided in the same health facility, though not necessarily in the same service point within the facility.

In November 2010, Rwanda EMTCT program adopted antiretroviral treatment (ART) for all pregnant and breastfeeding women (2010 WHO recommendation Option B). [7] Starting April 2012, the EMTCT program extended ART for all pregnant women beyond the breastfeeding period to life-long therapy (also known as Option B+). [7] From 2010 the EMTCT program included infant antiretroviral prophylaxis from birth to age 4–6 weeks. Table 1 compares Option A, B and B+. [7] Chi et.al. [8] reviewed updated literature in 2013 and confirmed the benefits of of all three options, however they note in their conclusion that “To better understand the downstream impact of the Option B+ approach, ‘real world’ program data are needed and must be carefully evaluated.” [8, pg 129] This report provides ‘real world’ data from the early Rwanda experience using Option B/B+.

**Table 1:** Comparison of PMTCT Options A, B and B+ [7, 8]

Timing	Option A		Option B		Option B+	
	Mother	Baby	Mother	Baby	Mother	Baby
Antepartum	ZDV from 14 weeks onward		Three drug ARV combination from 14 weeks until end of breastfeeding		Three drug ARV combination (from 1st HIV+ test) for life	
Intrapartum	Single-dose NVP					
Postpartum	1 week ZDV + 3TC	NVP for 6 weeks or until end of breastfeeding		NVP or ZDV until 6 weeks		NVP or ZDV until 6 weeks

*ZDV* zidovudine, *NVP* nevirapine, *3TC*, lamiduvine, *ARV* antiretroviral

This is the first report from a nationally representative study on the early (6–10 postnatal weeks) effectiveness of universal (Option B or B+) perinatal maternal ART on mother-to-child transmission of HIV to measure progress towards EMTCT targets in sub-Saharan Africa. The only other published national early EMTCT effectiveness studies have been from South Africa, [9, 10] which were done when South Africa was using WHO Option A and showed a 6 week transmission of 3.5% [7]. Other published early effectiveness studies of Option B or B+ have been only regional, not national studies with 10% MTCT found in Kenya [11] and 6.7% in Zimbabwe. [12, 13] Finally, one paper has been published on the national 18-month effectiveness of the Zimbabwe EMTCT program during the transition from Option A to Option B. [14] Therefore, this study adds substantially to the current available literature on national effectiveness of Option B or B+ at 6–10 postnatal weeks.

### Study objectives

The study objectives were to: i) to measure rates of early mother-to-child transmission (MTCT) of HIV at 6-weeks postpartum; ii) to estimate coverage of key EMTCT interventions and services (e.g. HIV testing, CD4 cell count testing, infant antiretroviral prophylaxis, counselling on infant feeding); iii) to estimate the association between MTCT rate and ART regimen, maternal background characteristics including CD4 cell count, maternal health care services and maternal and infant health status.

## Methods

A cross-sectional facility-based survey was conducted amongst mother/caregiver-infant pairs attending their 1st Diptheria-Tetanus-Pertussis (DTP1) immunization at six weeks postnatal. This study was conducted in public and faith-based primary health care facilities offering immunizations in all four Rwanda provinces and Kigali City. Health centers with and without an EMTCT program were selected in order to determine the national maternal and child HIV transmission rate. Routine data showed DTP1 coverage was 98.8%. [15] Facilities with high immunization coverage have been recognized for providing unique opportunities for cost-effectively measuring EMTCT program impact on early MTCT for EMTCT program evaluation. [16, 17] This methodology was first used by Rollins [18] and colleagues, and later was proven to be effective for measuring MTCT in South African. [9], The Rwanda EMTCT Evaluation protocol was adapted from the South African EMTCT Evaluation protocol and approved  by the National Institute of Statistics of Rwanda and by Rwanda’s National Ethics Committee.

### Sample

**Fig. 1:**
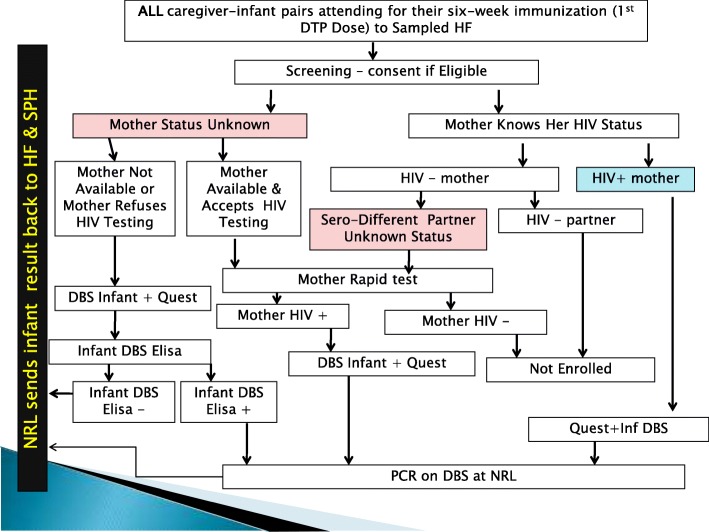
Testing and Enrollment Algorithm

Participants had to meet the following inclusion criteria: six-to-ten week old infants attending clinic for DPT1 immunization; written informed consent obtained from mother/caregiver; and mother known to be HIV-positive through self reporting, Mother Card or tested HIV-positive during DTP1 visit. For mothers who refused HIV testing, were dead or absent, but mother/guardian consented for the participation of the infant Dry Blood Spot (DBS) ELISA was performed to identify infant HIV exposure. The following were exclusion criteria: infants of mothers known HIV-negative with known HIV-negative partner; infants of mothers who tested HIV-negative to rapid test during the study; severely ill infants needing emergency medical care or urgent referral (e.g. severe vomiting, convulsions, lethargic or unconscious, or severe pneumonia or dehydration) (Fig. 1).

To determine the sample size for each health facility type, expected MTCT rates were calculated based on routine service delivery data for antenatal prevalence and coverage of EMTCT antiretroviral prophylaxis. Estimates of transmission rates for dual-therapy and no treatment are taken from routine service delivery data and previous findings on effectiveness of EMTCT in Rwanda at 18 months (best available data prior to this study) [19, 20].

For EMTCT facilities early (6–10 week) transmission was estimated as 8%, with precision of 2% required. For Non-EMTCT facilties early transmission was estimated as 25%, with 5% precision. A design effect of two was used to estimate sample size.

The median number of children immunised in the preceding 12 months from routine service delivery data was used to estimate expected number of children that could be recruited from a facility during the planned study duration. This was averaged for each stratum. The nonresponse rate considered was 5%. This information was used to calculate the number of facilities to be sampled to deliver the planned sample size of 2042 children (1412 in EMTCT Health Facilities and 630 in Non-EMTCT Health Facilities).

Sample size required in EMTCT health facilities were allocated proportionally (population proportionate-to-size) across strata (rural versus urban) based on observed 2009 strata immunization totals.

The primary sampling units were public health facilities and faith-based health facilities reportedly administering DTP1 doses in the Rwanda vaccination program report. While other public facilities do administer immunizations, primary level health facilities are the main locations for routine child health services. Data from the 2009 routine service delivery report [21] and the Health Information System database from the Ministry of Health were used to obtain the final sampling frame. The study was planned for data collection in 161 out of 412 (39%) primary health facilities over a period of seven months. Due to the small number of non-EMTCT health facilities available at study initiation (*n* = 36), all non-EMTCT health facilities were sampled.

### Data collection

Health facility nurses providing routine care were trained to recruit mothers/caregivers during immunization days. The nurses introduced the study and signed informed consent was obtained from each eligible mother/caregiver.

Data for the survey were gathered using a questionnaire. Several validated previously field-tested questionnaires were adapted. [9, 18] The questionnaire gathered information on maternal age, parity, socio-economic status (living arrangements; income), antenatal care (number of visits, gestation at first booking, HIV testing history; receipt of HIV test results; mother HIV test results; received EMTCT care during pregnancy and delivery; received infant feeding counseling; EMTCT prophylaxis taken (what and when); HIV-related care (CD4 count; on ART); birth information (birth weight; gestation; delivery type) and feeding practices during the past seven days. The questionnaire also documented current infant weight, immunizations, postnatal visits and illness since birth. The questionnaire was translated into Kinyarwanda (Rwanda national language). The questionnaire was tested in EMTCT sites not included in the study sample to test question flow and understanding by participants. Adjustments were made after the pilot (no significant changes necessary).

Trained health care providers collected blood samples. For mothers whose HIV status was unknown by the time of the study, blood samples were taken from consenting mothers and tested at the health center using Rwanda HIV rapid testing algorithm. Dry blood spot (DBS) from HIV-exposed children were tested for HIV using DNA PCR at the National Reference Laboratory of Rwanda. The National Reference Laboratory reported results back to the health facilities and the School of Public Health research team. The quality of the data collection process was checked on daily basis by nursing supervisors, trained laboratory staff and School of Public Health research team. Data entry, cleaning and validation was performed using EPIDATA and analysed using STATA SE 12.1.

### Data analysis

Formal survey analysis included the specification of the sampling stages and the finite number of primary sampling units involved. A weighted analysis was performed for Urban and Rural areas, as well as estimating the national prevalence. The final weights were calculated in 3 phases:

- Calculation of the Weight was based on the inclusion probability of a Health Facility: proportionate to the number of 1st DTP of the Health facility by Stratum
- Calculation of the Weight based on the Sample realization: number of observed (DBS taken and interview done)/number of expected
- Combination (multiply) of the two weights for the calculation of the final weight
- Truncation of the weight: every weight higher than 25 was rounded to 25.

For non-EMTCT health facilities, a weight of 1 was used since all of them were part of the study. All estimates are reported with 95% confidence limits.

Basic frequencies and 95% confidence intervals were used for descriptive analysis. All results are adjusted for clustering effects and weighted as appropriate for proportionate sampling methodology. Denominators for estimates are from those with reported data, missing not included.

Tests for association were carried out using chi-square tests or Fishers Exact tests. All results were adjusted for clustering. Multiple binary logistic regression analysis of predictors of HIV transmission at 6 weeks was conducted. Socio-demographic, health care services, EMTCT services and disease severity variables were examined as potential determinants of MTCT. Variables were retained for final multivariable models if they were either at least of marginal significance (*p*-value < 0.10) or played a confounding role (the variable had a noticeable impact > 10% change on the effect estimate).

## Results

Data collection was from July 2011 to June 2012. The sample realized was 1639 (82% of desired sample size). There was slightly lower sample realisation for Rural EMTCT facilities (892/1191, 75%), higher than expected sample realization for Urban EMTCT (655/234, 280%) and substantially lower sample realisation for Non-EMTCT sites (92/575, 16%). The sampling frame was adhered to for selected health facilities and allocated time spent was completed according to protocol, but the realization of participants was different from expected. This differential in expected participants experienced per strata was considered due to natural experience of women accessing EMTCT and accounted for during weighting at analysis stage.

**Fig. 2:**
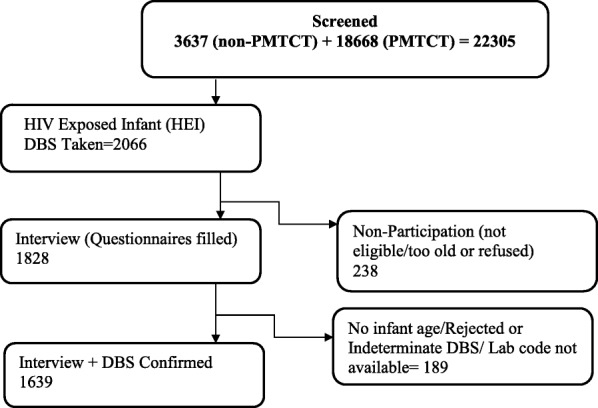
Study Profile

A total of 22,305 infants were screened (using a combination of: maternal interview, maternal HIV rapid testing for mothers who reported HIV status unknown, or HIV-negative mother in a discordant couple relationship). 2066 were identified as HIV-exposed infants and a DBS sample taken. Of these, 1828 met enrollment eligibility criteria, consented to study participation and were interviewed. 189 DBS were rejected by the lab or had indeterminant results, leaving 1639 subjects with both interview questionnaire completed and DNA PCR result available for analysis (Fig. 2).

The average age of the mothers was 29.5 years (SE 0.17) and the majority (72.5%) had primary school education. The majority were married (31.3%) or cohabitating (47.9%). The main building material of the floor was natural/other (68.1%), source of drinking water was a public tap (49.2%), use of a pit latrine (97.5%), and use of firewood/straw/dung/other as primary cooking fuel (72.5%). Almost one in five mothers (18.9%) reflected some food insecurity (i.e. ran out of food in the past year) and the majority had fewer assets (61.5% had 1–3 assets such as a radio, phone, etc.). Just under half of the mothers were employed (47.2%). The majority (81.1%) were multiparas and half (50.0%) had planned pregnancies.

Twenty-six infants were diagnosed HIV-positive on DNA PCR. The overall weighted MTCT rate was 1.58% (95% CI 1.05–2.38%). The rate was 1.28% (95%CI 0.71–2.30%) in rural areas and 2.06% in urban areas (95%CI 1.17–3.60%). The difference between the two areas was not statistically significant (*p* = 0.24).

**Fig. 3:**
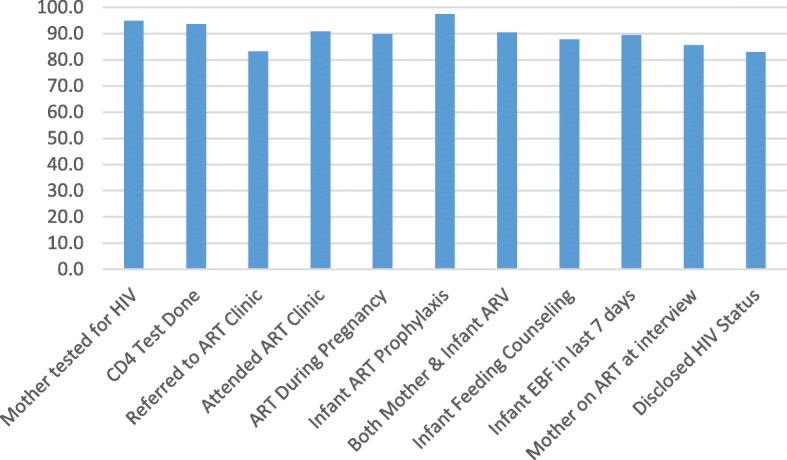
Rwanda Elimination of Mother to Child Transmission Programme (EMTCT) Cascade

Figure 3 shows results for EMTCT service indicators coverage in HIV-positive mothers and HIV-exposed infants. In all health centers, most of the key EMTCT indicators such as HIV testing, CD4 count, and maternal ART during pregnancy were above 90%. Referral to ART clinic, disclosure of HIV status and infant feeding counseling were below 90% but above 80%. 97.4% (95.8–98.4) of infants received antiretroviral prophylaxis. 91.1% (88.7–93.0) of HIV-positive mothers were on ART during pregnancy and 90.4% (87.9–92.3) of both mothers and infants received antiretroviral treatment/ prophylaxis per protocol. Exclusive breastfeeding in the last/previous 7 days was 88.1% (85.6–90.3) and coverage of maternal ART at six weeks was 85.6% (82.9–88.0).

In univariable analyses, socio-demographic factors significantly increasing the risk of MTCT (Table 2) included younger maternal age, marital status and having a flush toilet. Cohabitating was a protective factor reducing the risk of MTCT.

**Table 2:** Univariable Analysis of Infant HIV Infection by Socio-Demographic Factors

Variable	Answer (#)*	MTCT %	95%CI	*p*-value
Infant sex	Female (826)	1.8	1.1–3.0	0.462
	Male (813)	1.4	0.8–2.3	
Age of mother	35+ (399)	1.3	0.4–3.8	0.003
	25–34 (809)	0.9	0.4–1.8	
	15–24 (360)	3.6	2.3–5.7	
Education of mother	None (273)	2.1	0.9–4.9	0.308
	Primary (1145)	1.2	0.7–2.1	
	Secondary+ (183)	3.5	1.3–8.7	
Marital status of mother	Married (508)	2.5	1.1–5.5	0.046
	Co-Habitating (797)	0.6	0.2–1.2	
	Single/Other (324)	1.9	1.1–3.2	
Main building material of the floor	Natural/Other (1629)	2.7	1.4–5.1	0.334
	Rudimentary (152)	1.2	0.2–5.8	
	Finished (383)	1.3	0.7–2.2	
Main source of drinking water	Tap in House/Yard (154)	2.3	1.0–5.2	0.696
	Public Tap (859)	1.6	0.9–2.8	
	Well (covered or uncovered) (469)	1.1	0.4–3.1	
	Spring/River/Other (146)	2.3	0.8–6.6	
Type of toilet	Flush (16)	10.2	2.2–36.6	0.004
	Pit Latrine (1580)	1.5	1.0–2.4	
	None/Bush/Other (31)	0	–	
Main source of cooking fuel	Firewood/Dung/ Other (1133)	1.2	)0.7–2.1	0.071
	Elec/Gas/Kerosene/ Charcoal (497)	2.7	1.6–4.5	
Depletion of food in past 12 months	Never ran out of food (1320)	1.9	0.8–4.3	0.717
	Ran out of food (299)	1.5	0.9–2.5	
Number of Pregnancies	One (309)	2.3	1.2–4.4	0.312
	2 or more (1295)	1.4	0.9–2.4	
Planned Pregnancy	Not Planned (767)	1.2	0.7–2.1	0.079
	Planned (794)	2.0	1.2–3.3	
Religion	Catholic (662)	1.9	1.0–3.4	0.518
	Other Christian (739)	1.3	0.7–2.3	
	Other Religion (223)	1.9	1.0–3.5	
Rural or Urban	Rural (984)	1.3	0.7–2.3	0.268
	Urban (655)	2.1	1.2–3.6	
Source of income (can indicate multiple sources)	Self-Employed (744)	2.2	1.3–3.8	0.115
	Other Source (970)	0.9	0.5–1.6	
	None (96)	1.5	0.3–7.3	
Composite Asset Indicator (Electricity, Radio, TV, Phone, Refrigerator, Bicycle, Motorcycle/Scooter, Car)	0 assets (507)	1.3	0.6–2.9	0.323
	1–2-3 assets (1000)	1.5	0.8–2.7	
	4–5-6 assets (126)	3.5	1.7–7.5	

*Totals may not add to 100% due to missing

Multiple EMTCT and maternal and child health service factors were significantly associated with MTCT risk (Table 3) reflecting the impact of missed opportunities in the EMTCT cascade even in the context of high EMTCT service coverage. HIV-exposed infants of mothers who were not referred to the ART clinic, or started ART during pregnancy or after delivery compared to starting before pregnancy, were risk factors for MTCT. HIV-exposed infants whose mothers tested HIV-negative or were not tested during pregnancy or not taking ART during pregnancy were at increased risk of MTCT, as was starting infant antiretroviral prophylaxis after 72 h and incomplete antiretroviral treatment/prophylaxis (lack of anitretrovirals in either mother or baby or both), however these variables did not reach statistical significance (*p* < 0.05). Lack of infant feeding counseling or disclosure of HIV status also increased MTCT risk. Late attendance at antenatal care (3rd trimester) by the mother was a risk factor. Not testing women for syphilis during antenatal care was a risk factor for MTCT, but this variable did not reach statistical significance (p < 0.05).

**Table 3:** Univariable Analysis of Infant HIV Infection by EMTCT Service and Other Health System & Maternal/Infant indicators

Variable	Answer (#)*	MTCT %	95%CI	*p*-value
EMTCT Site	Yes (1547)	1.6	1.1–2.5	0.097
	No (92)	0.7	0.2–2.4	
HIV Test Results written on Card	Yes (1516)	1.6	1.0–2.5	0.0504
	No (81)	0.5	0.1–1.9	
Mother HIV testing during pregnancy (in Negative or Unknown at beginning of pregnancy)	Yes (1615)	1.6	1.0–2.4	0.6220
	No (24)	2.4	0.6–10.0	
Mother HIV status known during pregnancy	Tested HIV+ during pregnancy (1280)	1.2	0.7–2.1	0.051
	Tested HIV Negative during pregnancy (162)	3.5	1.7–7.0	
	Not tested during pregnancy (177)	2.7	1.4–5.1	
Mother CD4 Count done	CD4 done (1505)	1.6	1.0–2.4	0.432
	CD4 not done (92)	2.7	1.07.4	
Mother referred to ART clinic	Yes referred (1316)	1.1	0.6–2.0	0.034
	Not referred (242)	3.2	1.9–5.6	
CD4 level	> 350 (275)	2.2	1.1–4.5	0.328
	<=350 (1075)	1.4	0.7–2.5	
Mother visited ART clinic	Yes (1467)	1.6	1.1–2.5	0.637
	No (107)	1.2	0.3–4.6	
Maternal took ART/ARV during pregnancy	Yes (1474)	1.3	0.8–2.1	0.051
	No (120)	4.9	2.4–9.8	
Mother timing of initiation of ART	Prior to pregnancy (637)	0.4	0.2–0.9	0.012
	During Pregnancy (686)	1.9	1.1–3.4	
	After Delivery (44)	8.6	2.5–26.2	
Infant ARV Prophylaxis Initiation	Nevirapine within 72 h (1149)	1.2	1.0–2.4	0.062
	Nevirapine not within 72 hoursbut within 6 weeks/no (430)	2.7	1.6–4.5	
Mother-Baby ART/ARV	Mother & Baby Received (1422)	1.3	0.8–2.2	0.056
	Not Mother-Baby ART/ARV (either mother or baby or both no treatment) (128)	4.7	2.3–9.3	
Infant Cotrimoxazole	Yes (639)	1.1	0.5–2.7	0.217
	No (965)	2.0	1.3–3.2	
Infant Feeding Counselling received	Yes (1420)	1.1	0.6–2.0	0.024
	No (194)	3.6	2.1–6.1	
Infant Feeding exclusive breastfeeding in last 24 h	Yes (1439)	1.5	0.9–2.3	0.399
	No (196)	2.4	1.0–5.7	
Any Breastfeeding	Yes (1429)	1.5	1.0–2.4	0.535
	No (210)	2.1	0.9–5.1	
Disclosed status	Yes (1340)	1.1	0.7–1.9	0.006
	Nov (252)	4.1	2.4–6.8	
Perceived Discrimination	Yes (212)	2.7	1.2–6.0	0.255
	No (1372)	1.5	0.9–2.3	
Antenatal Care	0 visits (17)	11.3	3.5–30.9	0.130
	1 or more visits (1586)	1.5	1.0–2.3	
Gestational Age started antenatal care	< 27 Weeks (1093)	1.0	0.5–2.0	0.049
	> = 27 Weeks (546)	2.7	1.5–4.6	
Syphilis Screen during pregnancy	Yes (1264)	1.4	0.9–2.3	0.062
	No (237)	3.0	1.8–5.2	
Tuberculosis Screen during pregnancy	Yes (602)	1.9	1.0–3.6	0.458
	No (952)	1.4	0.8–2.4	
Place of Delivery	Facility (1525)	1.5	0.9–2.2	0.257
	Home/Other (98)	3.5	1.3–9.1	
Method of Delivery	Vaginal (1343)	1.4	0.9–2.2	0.897
	Caesarean Section (237)	1.5	0.6–4.0	
Delivered by	Doctor (310)	1.4	0.6–3.3	0.449
	Midwife/Nurse (1217)	1.4	0.9–2.4	
	Traditional Birth Attendant/Other (80)	4.2	1.6–10.6	
Low Birth Weight	Yes (85)	2.2	1.0–4.9	0.467
	No (1533)	1.5	1.0–2.4	
Preterm	Yes (41)	3.5	1.3–9.4	0.349
	No (1598)	1.5	1.0–2.4	

*Totals may not add to 100% due to missing

Three multivariate models were created to assess independent effects on MTCT. Variables in univariable analysis (Tables 2 and 3) with a *p*-value < 0.10 were included in the first two multivariable models. The third model combined significant variables from these intial two models for a final model examining both socio-demographic and EMTCT service factors. A backward step-wise approach was used to generate the final model (Table 4). Due to concerns about statistical power as a result of small number of events (*n* = 26) and potential co-linearity between certain variables (e.g. referral for maternal ART and receipt of maternal ART, measures of maternal and infant antiretroviral treatment/prophylaxis) only the following variables were included in the full model: Maternal HIV testing status; Mother ART clinic referral; Infant Feeding Counseling; HIV Status Disclosure; Late Antenatal Care – 3rd Trimester; Syphilis testing during pregnancy; Delivery attendant; Preterm delivery; Both Mother and Baby getting antiretroviral treatment/prophylaxis; Timing of maternal ART initiation; Timing of infant Nevirapine initiation.

**Table 4:** Final Multivariable Model examining socio-demographic and EMTCT service variables as predictors of MTCT

Variables	Odds ratio	Std Err.	t	Pr(T > t)	95% Confidence Interval	
	5.53	2.53	3.74	< 0.001	2.24	13.70
Mother timing of initiation of ART during pregnancy (Ref: 1=prior to pregnancy)	2.03	1.18	1.22	0.226	0.64	6.44
Mother timing of initiation of ART after delivery (Ref: 1=prior to pregnancy)	236.54	267.37	4,84	< 0.001	25.26	2213.05
No Disclosure (Ref: Disclosure)	6.19	1.87	6.05	< 0.001	3.41	11.24
Gestational Age started antenatal care > = 27 weeks (Ref:< 27 weeks)	1.65	0.73	1.12	0.264	0.68	3.97
No Syphilis Screen during pregnancy (Ref: Yes)	2.87	0.90	3.35	0.001	1.54	5.35
Full Term (Ref: Preterm)	0.24	0.09	−3.95	< 0.001	0.12	0.50
Infant ARV Prophylaxis Initiation within 6 weeks (Ref:Nevirapine within 72 h)	5.01	1.99	4.05	< 0.001	2.28	11.02
Both Mother & Baby got ART/ARV (Ref: either mother or baby or both no ART/ARV)	0.01	0.18	−3.40	0.001	0.001	0.17
Mother age > 25 (Ref: <=25 years old)	0.33	0.13	−2.54	0.012	0.14	0.78
Source of income Self-employed (Ref: None)	2.78	1.62	1.75	0.082	0.88	8.82
Type of toilet Pit (Ref: Flush)	0.12	0.07	−3.92	< 0.001	0.04	0.35

A large number of risk factors remained significant in the final model. Receipt of maternal ART and infant antiretroviral prophylaxis according to Rwanda EMTCT protocol was a highly significant protective factor against MTCT (OR 0.01; 95%CI 0.001–0.17), while variables suggesting inadequate antiretroviral treatment/prophylaxis were all significant risk factors (No referral for ART, started of ART after delivery, and start of infant Nevirapine after 72 h). Maternal age older than 25 years was protective (OR 0.33; 95%CI 0.14–0.78). Factors suggesting higher socio-demographic status were risk factors for MTCT (flush toilet, mother self-employed (borderline significant). No disclosure of HIV status, no syphilis testing during pregnancy and preterm birth were also significant risk factors.

## Discussion

The Rwanda rate of early transmission of HIV from mother-to-child of 1.58% (95% CI 1.05–2.38%) showed a reduction from the 2.6% at 6 weeks reported by routine service delivery data in 2008, though this earlier data from the routine EID program may have reflected selection bias. [21] It also compares favourably to 3.5% documented in South Africa which was using a combination of dual-therapy and maternal ART (WHO Option A). [9] Compared to regional studies of Option B or B+, this rate compares favorably to 10% MTCT found in Kenya and 6.7% in Zimbabwe. [12, 13] The Rwanda national target is 2% at 18 months.

The coverage in the EMTCT Cascade is seen to be high with all indicators above 80% and many over 90%. (Fig. 3) The primary indicator for EMTCT program coverage is mothers who received ART during pregnancy, this showed a coverage of 91.1%, which is just above the Rwanda 2012 national target of 90%. [6] When both mothers and infants are considered 90.4% of mothers and babies received the recommended antiretroviral treatment/prophylaxis. Over 90% coverage of recommended antiretroviral treatment/prophylaxis during pregnancy is also consistent with UNAIDS targets for global elimination of MTCT, however the drop to 85.6% at 6–10 weeks is a concern as it is below the UNAIDS target for ART during breastfeeding of 90%. [10] The coverage figures for mothers and babies combined compare favorably to other studies, such as 65% in Zimbabwe, [13] 26% in rural Kenya, [11]. These figures also suggest approximately 10% missed opportunities for antiretroviral therapy/prophylaxis per Rwandan protocol in mother-baby pairs. These missed opportunities represented a significant risk for increased MTCT (Table 4), which is consistent with other reports in the literature [9–11].

Several socio-demographic factors showed in univariable analysis to increase MTCT risk, however, only flush toilet and mother self-employed remained significant or borderline significant, respectively, in final multivariable analysis (Table 4). This finding suggests that higher socio-economic mothers seem more at risk of MTCT. Bucagu et al. [22] did not find any association of socio-economic status to MTCT in one health center in Rwanda, however this report was limited in scope and size. While some studies suggest the poor face more barriers to access EMTCT services [23, 24], the findings in this study are consistent with findings from a Nigerian study [25] which found that higher income mothers were less likely to access EMTCT services. Further studies on the relationship of socio-economic status, utilization of EMTCT services and MTCT in Rwanda are needed to allow better targeting of services across populations.

Teen and young mothers (< 25 years old) also showed a higher risk for mother-to-child transmission in the final multivariable analyses. This result is consistent with the Bucagu study which found a small but non-significant increased risk for women age less than 24 [22] and is also consistent with the Nigeria study which found a strong association between teenage mothers and MTCT risk [25]. Younger mothers are theorized to utilize EMTCT services less than older women [25] so this issue is important in the context of the Rwandan EMTCT program.

Lack of disclosure of HIV status showed an almost 7-fold increase in MTCT in the multivariable analysis. More than half (53–65%) of people in the Rwanda DHS indicated an accepting attitude towards persons with HIV/AIDS, [15] and a study of Rwandans with HIV showed limited perceived discrimination [26]. However, lack of disclosure and stigma may impact on both treatment adherence and safe infant feeding. Stigma and disclosure to family and friends of HIV status have consistently been seen to impact EMTCT care and treatment adherence in the literature [22, 26–29].

Two antenatal care variables were significant risk factors for MTCT, antenatal care started after 27 weeks gestation and no maternal syphilis test during pregnancy. Not having a syphilis test remained significant in the final model. This association was also seen in a South African cohort study, which theorized that syphilis testing may be a marker for quality of antenatal care [30]. More recent policy analyses suggest the need for integration across HIV and Syphillis elimination programs to improve antenatal quality of care [31]. Preterm birth was a risk factor for MTCT which is consistent with the literature [30].

Limitations of the current design must be recognized. The data were facility-based using infants presenting for immunization. In their 2012 publication ‘*A short guide on methods: measuring the impact of national EMTCT programmes: towards the elimination of new HIV infections in children by 2015 and keeping their mothers alive, WHO* recognizes this methodology as one of several available to assess EMTCT impact. [17] This document notes the following pros and cons for this method: “in settings with high immunization coverage, real time data can be captured to inform population-level transmission and early infant HIV infection; relatively quick to undertake and can be repeated to provide trend data, especially if a modest amount of additional data is collected at the same time; also provides results for infants of mothers who did not attend antenatal clinic or receive EMTCT care; however this design  misses children who have died before immunization” [17,pg9].

In Rwanda the 6 week immunization visit has high attendance nationally (98.8%) meeting the condition for high immunisaiton coverage as suggested by WHO. In addition, the socio-demographic factors in this study were similar to those in the Rwanda DHS completed the year prior to the study. [15] Identification and referral of eligible infants to the clinic was also done during the immunization outreach strategy program in villages to maximize the chance of having all eligible infants. Infants who did not come for immunization, had severe illness or had already died by 6 weeks of age were not included in the sample suggesting a possible under-estimation of prevalence. Also, there were differences in the expected sample size per sampling strata (e.g. urban/rural and EMTCT/Non-EMTCT). These differentials may have existed as there were fewer Non-EMTCT sites due to rapid EMTCT program scale-up, and clients may have traveled to urban areas and EMTCT clinic sites in expection of more comprehensive services. These differences across strata were accounted for by weighting during statistical analyses. There is always potential for recall bias, however the relatively short recall period of 6 weeks minimizes this potential bias. Social desirability bias and fear of disclosure and stigma may cause mothers to either over or under-report participation in the EMTCT program. Confidentiality was assured and discussed as part of the informed consent process and a private place was secured for conducting interviews. In addition, data collectors were routine clinic nurses who had full access to maternal and newborn records to identify possible HIV-exposed infants. Established sensitivity and specificity of the HIV testing were operational, but should have been minimized through proper handling of specimens and assurance of laboratory quality control. The study did not include a measure of daily adherence to ART only regimen. In addition, national averages could hide subnational disparities not addressed in this study and suggest a need for further investigations on equity in the EMTCT program.

## Conclusions

The low rate of MTCT of 1.58% achieved in Rwanda is a clear indication that new HIV infections in children can reach elimination targets. To reduce the the MTCT rate futher, the program must begin to address existing missed opportunities, particularly with regard to coverage of maternal ART and infant antiretroviral prophylaxis. Integration of EMTCT within the maternal and child health care platform [32], enhanced early treatment with ART and retention in care, starting antenatal care early and continuing antenatal care are some of the strategies to minimize the risks of missed opportunities. In addition, young, single mothers appear to be at higher risk suggesting the need for targeting this population.

## Abbreviations

ART: antiretroviral therapy
ARV: antiretroviral (prophylaxis)
CD4: cluster of differentiation 4, a type of lymphocite
DBS: dry blood spot
DNA PCR: deoxyribonucleic acid polymerase chain reaction
DPT1: diptheria, polio and tetanus vaccine, given as first dose
EMTCT: elimination of mother-to-child transmission of HIV
HIV: human immunodefieciency virus
MTCT: mother-to-child transmission of HIV, also know as vertical transmission of HIV
UNAIDS: The Joint United Nations Program on HIV/AIDS
WHO: World Health Organization
